# Effect of dexmedetomidine supplementation for thoracoscopic surgery: a meta-analysis of randomized controlled trials

**DOI:** 10.1186/s13019-022-01803-z

**Published:** 2022-04-06

**Authors:** Chengjun Song, Quan Lu

**Affiliations:** 1 Department of Cardiothoracic Surgery, Fenghua District People’s Hospital of Ningbo, Zhejiang, China; 2 Department of Anesthesiology, Fenghua District People’s Hospital of Ningbo, Zhejiang, China

**Keywords:** Dexmedetomidine, Thoracoscopic surgery, Analgesic efficacy, Randomized controlled trials

## Abstract

**Introduction:**

The efficacy of dexmedetomidine supplementation for thoracoscopic surgery remains controversial. We conduct a systematic review and meta-analysis to explore the impact of dexmedetomidine for thoracoscopic surgery.

**Methods:**

We have searched PubMed, EMbase, Web of science, EBSCO, and Cochrane library databases through September 2020 for randomized controlled trials (RCTs) assessing the effect of dexmedetomidine supplementation on thoracoscopic surgery. This meta-analysis is performed using the random-effect model.

**Results:**

Six RCTs involving 510 patients are included in the meta-analysis. Overall, compared with control group for thoracoscopic surgery, dexmedetomidine supplementation results in significantly reduced pain scores (SMD = − 1.50; 95% CI = − 2.63–− 0.37; P = 0.009), anesthetic consumption (SMD = − 3.91; 95% CI = − 6.76–− 1.05; P = 0.007), mean heart rate (SMD = − 0.41; 95% CI = − 0.65–− 0.18; P = 0.0007), and the risk ratio (RR) of ICU stay (RR = 0.39; 95% CI = 0.19–0.80; P = 0.01), but showed no obvious effect on mean blood pressure (SMD = − 0.07; 95% CI = − 0.45–0.31; P = 0.72) or hospital stay (SMD = − 0.61; 95% CI = − 1.30–0.08; P = 0.08).

**Conclusions:**

Dexmedetomidine supplementation can substantially improve the analgesic efficacy for thoracoscopic surgery.

**Supplementary Information:**

The online version contains supplementary material available at 10.1186/s13019-022-01803-z.

## Introduction

Thoracoscopic surgery is widely used to treat various diseases such as esophageal cancer and lung cancer. It results the smaller incision, less pain and inflammatory response, reduced recovery times compared to traditional surgery [1–3]. The pain commonly occurs after the surgery, and negatively affects the postoperative recovery. Various analgesic regimens have developed for the pain management after thoracoscopic surgery, and they mainly include pharmacologic and regional interventions (e.g. nerve block) [4–7].

Dexmedetomidine, a short-acting α2 -adrenoceptor agonist, is reported to provide the sedation and analgesia for various surgeries [8, 9]. Studies demonstrated that dexmedetomidine attenuated surgical stress responses in patients undergoing surgery, and is effective and safe to improve the analgesic efficacy when serving as an adjunctive analgesic [10, 11]. Previous trials demonstrated that dexmedetomidine had opioid-sparing properties, maximized pain relief and minimized analgesic-related side effects [12–14].

However, the efficacy of dexmedetomidine supplementation for thoracoscopic surgery has not been well established. Recently, several studies on the topic have been published, and the results were conflicting [7, 15–17]. For instance, two studies reported that dexmedetomidine supplementation could significantly reduce postoperative pain scores for thoracoscopic surgery [16, 18], but another study found no benefits to pain control after using dexmedetomidine supplementation for thoracoscopic surgery [7]. With accumulating evidence, we therefore perform a systematic review and meta-analysis of RCTs to investigate the analgesic efficacy of dexmedetomidine supplementation for thoracoscopic surgery.

## Materials and methods

Ethical approval and patient consent are not required because this is a systematic review and meta-analysis of previously published studies. The systematic review and meta-analysis are conducted and reported in adherence to PRISMA (Preferred Reporting Items for Systematic Reviews and Meta-Analyses) [19].

### Search strategy and study selection

Two investigators have independently searched the following databases (inception to September 2020): PubMed, EMbase, Web of science, EBSCO, and Cochrane library databases. The electronic search strategy was conducted using the following keywords: “dexmedetomidine”, and “thoracoscopic” or “thoracoscopy”. We also check the reference lists of the screened full-text studies to identify other potentially eligible trials.

The inclusive selection criteria are as follows: (i) patients underwent thoracoscopic surgery; (ii) intervention treatments were intravenous dexmedetomidine supplementation versus no dexmedetomidine; (iii) study design was RCT.

### Data extraction and outcome measures

We have extracted the following information: author, number of patients, age, sex, body mass index, American Society of Anesthesiologists (ASA) and detail methods in each group. The ASA Physical Status Classification System is the most widely used system globally to describe a patient’s preoperative medical condition. The four categories (P1–P4) in the classification have changed little since they were first proposed in 1941 [20]. Data were extracted independently by two investigators, and discrepancies are resolved by consensus. We also contacted the corresponding author to obtain the data when necessary.

The primary outcome was pain scores. Secondary outcomes included analgesic consumption, mean heart rate and blood pressure, ICU stay, and hospital stay.

### Quality assessment in individual studies

Methodological quality of the included studies is independently evaluated using the Jadad scale [21]. There are 3 items for Jadad scale: randomization (0–2 points), blinding (0–2 points), dropouts and withdrawals (0–1 points). The score of Jadad Scale varies from 0 to 5 points. An article with Jadad score ≤ 2 is considered to be of low quality. If the Jadad score ≥ 3, the study is thought to be of high quality [22].

### Statistical analysis

We estimate the standard mean difference (SMD) with 95% confidence interval (CI) for continuous outcomes (pain scores, analgesic consumption, mean heart rate and blood pressure, and hospital stay) and relative risk (RR) with 95% CI for dichotomous outcomes (ICU stay). The random-effects model was used regardless of heterogeneity. Heterogeneity was reported using the I^2^ statistic, and I^2^ > 50% indicated significant heterogeneity [23]. Whenever significant heterogeneity was present, we searched for potential sources of heterogeneity via omitting one study in turn for the meta-analysis or performing subgroup analysis. Publication bias was not evaluated because of the limited number (< 10) of included studies. All statistical analyses were performed using Review Manager Version 5.3 (The Cochrane Collaboration, Software Update, Oxford, UK).

## Results

### Literature search, study characteristics and quality assessment

A detailed flowchart of the search and selection results was shown in Additional file 1: Fig. S1. 239 potentially relevant articles are identified initially. Finally, six RCTs that meet our inclusion criteria are included in the meta-analysis [7, 15–18, 24].

The baseline characteristics of the six eligible RCTs in the meta-analysis were summarized in Table 1. The six studies were published between 2016 and 2020, and the total sample size was 510. Dexmedetomidine was used before the anesthesia [7, 15, 18, 24], or during surgery [16, 17].

**Table 1:** Characteristics of included studies

No	Author	Dexmedetomidine group						Control group									
		Number	Age (years)	Sex(male/female)	Body mass index (kg/m2)	ASA (I/II/III)	Methods	Number	Age (years)	Sex(male/female)	Body mass index (kg/m2)	ASA (I/II/III)	Methods	Surgery type	Combined anaesthetics	Outcomes	Jada scores
1	Wang 2020	46	56.78 ± 12.81	17/29	22.09 ± 3.22	7/39/0	dexmedetomidine 0.8 μ g/kg administered for 10 min before anesthesia	44	60.48 ± 12.58	22/22	22.89 ± 2.85	10/34/0	placebo	video-assisted thoracoscopic lung lobectomy	fentanyl, propofol and isoflurane	pain score (numeric rating scale), heart rate, blood pressure	3 (minus 2, undescribed blindness)
2	Kim 2019	60	63 [58–68], median [interquartile range]	28/32	24 ± 3	18/42/0	dexmedetomidine started after inducing anesthesia and continued until the end of surgery at a fixed dose (0.5 ug/kg/h)	60	59 [56–65]	30/30	23 ± 5	20/40/0	placebo	thoracoscopic lung resection surgery	sevoflurane	pain score (numeric rating scale), analgesic consumption (opioids), ICU stay	5
3	Wu 2018	30	59.0 ± 8.8	15/15	-	2/14/14	0.5 ug/kg/h dexmedetomidine through the surgery	30	58.7 ± 10.1	16/14	-	1/18/11	placebo	thoracoscopic surgery	fentanyl, propofol and sevoflurane	heart rate, blood pressure, ICU stay and hospital stay	4 (minus 1, unclear blindness)
4	Wang 2016	40	54.25 ± 9.98	20/20	21.93 ± 2.12	-	0.5 μ g/kg, dexmedetomidine diluted to 20 mL with physiologic saline and infused for 10 min intravenously before the surgery	40	55.63 ± 11.20	20/20	22.10 ± 2.13	-	no dexmedetomidine	video-assisted thoracoscopic lobectomy	oxycodone, propofol, fentanyl and sevoflurane	analgesic consumption (oxycodone dose), heart rate, blood pressure	5
5	Lee 2016	50	62.0 ± 10.5	26/24	23.6 ± 0.4	0/37/13	dexmedetomidine 1.0 ug/kg for 20 min before the termination of surgery	50	62.0 ± 11.5	23/27	23.6 ± 0.4	0/42/8	placebo	video-assisted thoracoscopic surgery for lung cancer	propofol, remifentanil, desflurane and fentanyl	pain score (numeric rating scale), analgesic consumption (opioids), ICU stay	5
6	Lee 2016 (2)	25	68.4 ± 6.4	12/13	22.3 ± 2.7	0/11/14	dexmedetomidine at an initial loading dose of 1.0 ug/kg over 10 min followed by a maintenance dose of 0.5 ug/kg/h during the surgery	25	69.4 ± 8.7	11/14	22.7 ± 2.1	0/12/13	placebo	thoracoscopy for lung resection	propofol, remifentanil, and sevoflurane	heart rate, blood pressure, ICU stay and hospital stay	5

*ASA* American Society of Anesthesiologists

Among the six studies included here, three studies reported pain scores [15, 16, 18], three studies reported analgesic consumption [7, 16, 18], four studies reported mean heart rate and blood pressure [7, 15, 17, 18], three studies reported ICU stay [15, 17, 24], and three studies reported hospital stay [17, 18, 24]. Jadad scores of the six included studies vary from 3 to 5, and all six studies are considered to be high-quality ones according to quality assessment.

### Primary outcome: pain scores

**Fig. 1:**
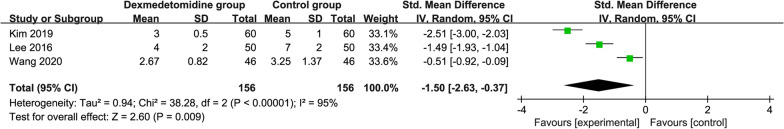
Forest plot for the meta-analysis of pain scores

This outcome data was analyzed with the random-effects model, and the pooled estimate of the three included RCTs suggested that compared to control group for thoracoscopic surgery, dexmedetomidine was associated with significantly reduced pain scores (SMD = -1.50; 95% CI = -2.63 to -0.37; P = 0.009), with significant heterogeneity among the studies (I^2^ = 95%, heterogeneity P < 0.00001) (Fig. 1).

### Sensitivity analysis

**Fig. 2:**
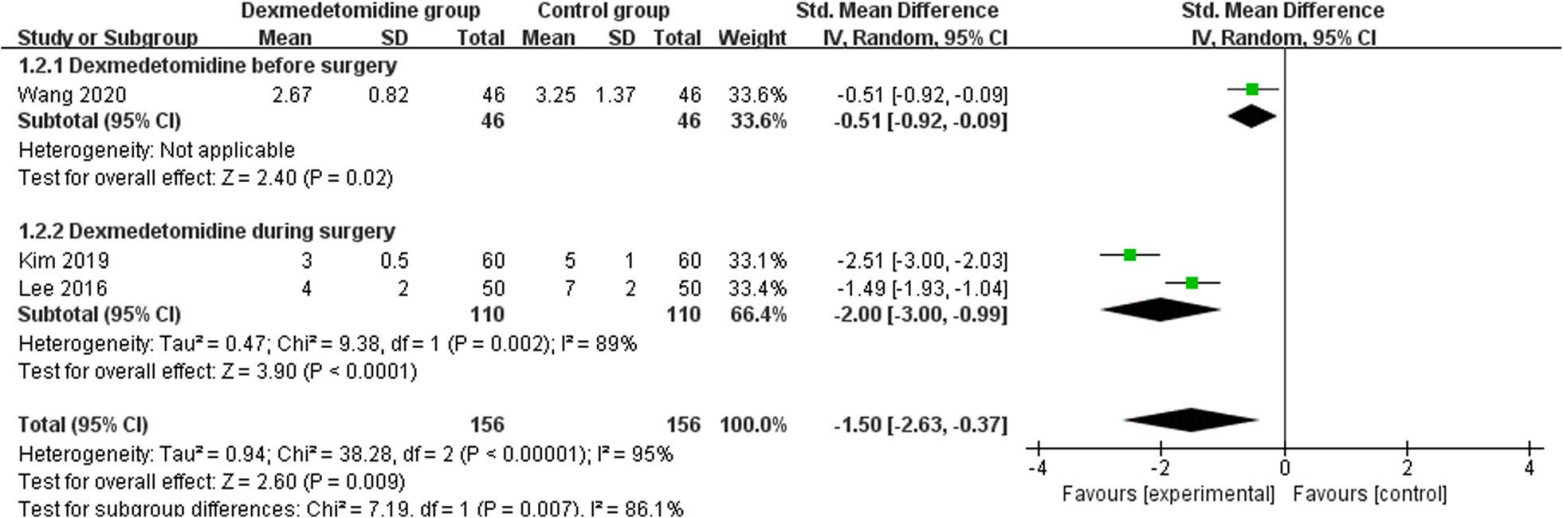
Subgroup analysis of pain scores based on dexmedetomidine supplementation before surgery versus during surgery

Significant heterogeneity is observed among the included studies for the primary outcomes, but there is still significant heterogeneity after when performing sensitivity analysis via omitting one study in turn to detect the heterogeneity (I^2^ ranging from 89 to 97%). In addition, we perform the subgroup analysis based on dexmedetomidine supplementation before vs during surgery, but there is still significant heterogeneity (I^2^ = 89%). The results find that dexmedetomidine supplementation results in substantially reduced pain scores when administered before surgery (P = 0.02) and during surgery (P < 0.0001, Fig. 2).

### Secondary outcomes

**Fig. 3:**
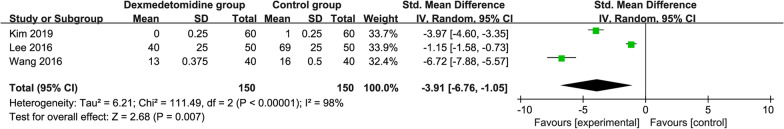
Forest plot for the meta-analysis of analgesic consumption

**Fig. 4:**
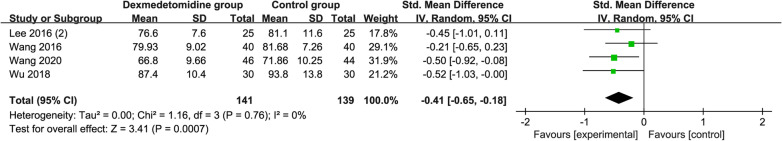
Forest plot for the meta-analysis of mean heart rate

**Fig. 5:**
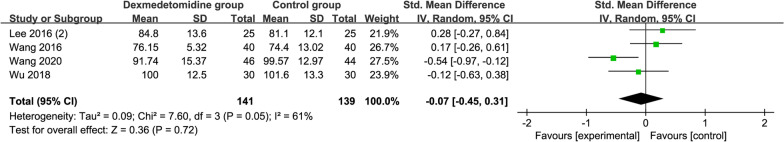
Forest plot for the meta-analysis of mean blood pressure

**Fig. 6:**
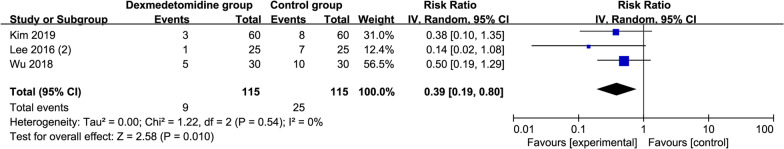
Forest plot for the meta-analysis of ICU stay

**Fig. 7:**
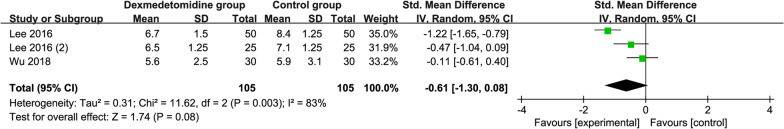
Forest plot for the meta-analysis of hospital stay

Compared to control group for thoracoscopic surgery, dexmedetomidine can significantly reduce anesthetic consumption (SMD = − 3.91; 95% CI = -6.76–− 1.05; P = 0.007; Fig. 3) and mean heart rate (SMD = − 0.41; 95% CI = − 0.65–− 0.18; P = 0.0007; Fig. 4), but has no important impact on mean blood pressure (SMD = − 0.07; 95% CI = − 0.45–0.31; P = 0.72; Fig. 5). In addition, dexmedetomidine was associated with the decrease in the RR of ICU stay (RR = 0.39; 95% CI = 0.19–0.80; P = 0.01; Fig. 6), but revealed no effect on hospital stay (SMD = − 0.61; 95% CI = − 1.30 to 0.08; P = 0.08; Fig. 7).

## Discussion

Thoracoscopic surgery has been widely used to treat lung cancer because of its minimally invasion, less postoperative pain and shortened hospital stay compared with open thoracotomy [25]. Postoperative pain management, particularly early postoperative pain, still remains a matter of concern for many anesthesiologists and these patients [26, 27]. Opioids are essential during surgery, and many methods are developed to reduce opioid consumption due to the side effects such as delayed recovery from general anesthesia, opioid-induced nausea, and respiratory depression [28, 29].

Intraoperative dexmedetomidine was reported to improve the effects of postoperative analgesia [30–32]. It showed analgesic, sedative and anxiolytic effects, and avoided respiratory depression and the inhibitory effect of sympathetic stimulation as an adjunct to general anesthesia [8]. Our meta-analysis included six RCTs and 510 patients. The results revealed that intravenous dexmedetomidine was associated with substantially reduced pain scores, anesthetic consumption, the RR of ICU stay and mean heart rate after thoracoscopic surgery, but showed no obvious influence on mean blood pressure or hospital stay.

In addition, dexmedetomidine benefited to maintain the stability of the cardiovascular system and decrease the stress response [10]. Intraoperative infusion of dexmedetomidine decreased both norepinephrine and epinephrine. Dexmedetomidine can decrease the release of catecholamines and has analgesic, anxiolytic, and hypnotic effects [33]. Regarding the sensitivity analysis, there is significant heterogeneity. Several reasons may account for the heterogeneity. Firstly, different doses and methods of dexmedetomidine supplementation may produce some bias. For instance, Dexmedetomidine was used before the anesthesia [7, 15, 18, 24] or during surgery [16, 17]. Secondly, dexmedetomidine was applied as the adjunct to different drugs such as oxycodone and sevoflurane, which may result in various analgesic effect. Thirdly, different operation procedures produces various pain intensity, which may affect the pooling results.

This meta-analysis has several potential limitations. Firstly, our analysis is based on only six RCTs, and three of them have a relatively small sample size (n < 100). Overestimation of the treatment effect was more likely in smaller trials compared with larger samples. Next, the doses, methods and combination of anesthetic drugs in included RCTs are different, which may have an influence on the pooling results. Finally, thoracoscopic surgeries are performed for various diseases and operation procedures.

## Conclusions

Dexmedetomidine benefits to improve the analgesic efficacy for thoracoscopic surgery.

## Supplementary Information

**Additional file 1: Figure S1.** Flow diagram of study searching and selection process.

## Data Availability

Not applicable.

## Abbreviations

RCTs: Randomized controlled trials
MDs: Mean differences
CIs: Confidence intervals
RRs: Risk ratios
