# Supplementary figures and images for: Effect of dexmedetomidine supplementation for thoracoscopic surgery: a meta-analysis of randomized controlled trials
Source: J Cardiothorac Surg. 2022 Apr 6;17:70. doi: 10.1186/s13019-022-01803-z (PMC8985285; doi:10.1186/s13019-022-01803-z)

**
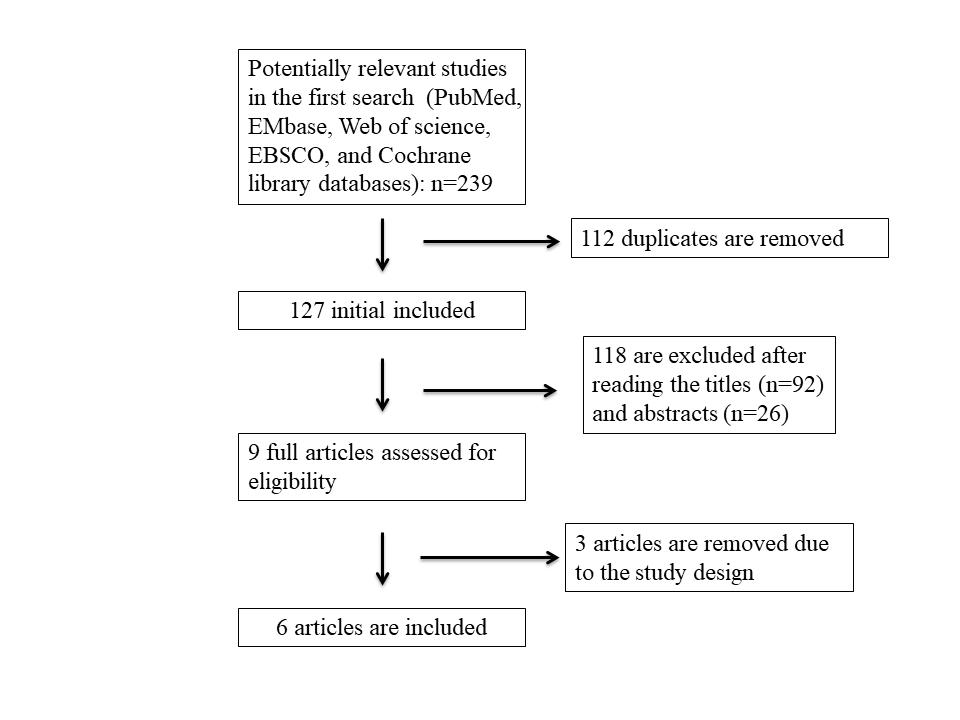
**

**Additional file 1: figure S1** Flow diagram of study searching and selection process.

Supplement: Supplementary file 1 — Additional file 1: Figure S1. Flow diagram of study searching and selection process. [file 13019_2022_1803_MOESM1_ESM.docx]
